# Supplementary material for: Characterisation of ACP5 missense mutations encoding tartrate-resistant acid phosphatase associated with spondyloenchondrodysplasia
Source: PLoS One. 2020 Mar 26;15(3):e0230052. doi: 10.1371/journal.pone.0230052 (PMC7098635; doi:10.1371/journal.pone.0230052)

Original scans of raw western blots (PONE-D-19-20746R1)

Figure 7 Wild type vs DC 1X

1. From lanes 2 to 4 medium (left three lanes) and lanes 2 to 4 of cells (right three lanes)

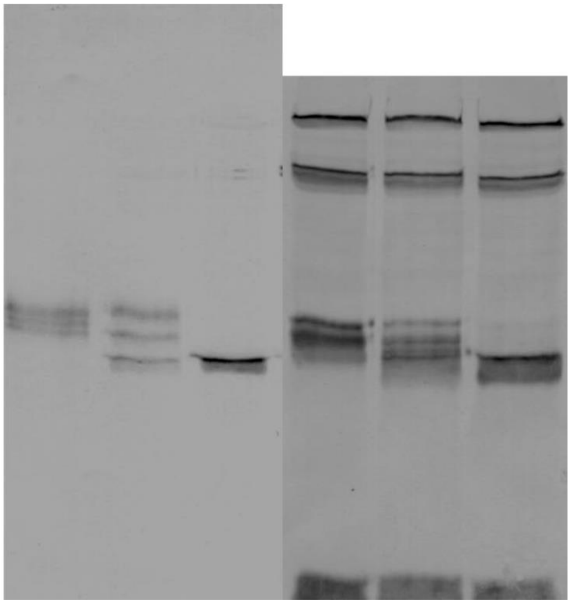

2. From lanes 5 to 10

Medium

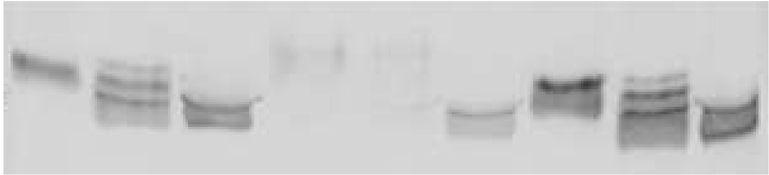

3. From lanes 11 to 19

Medium

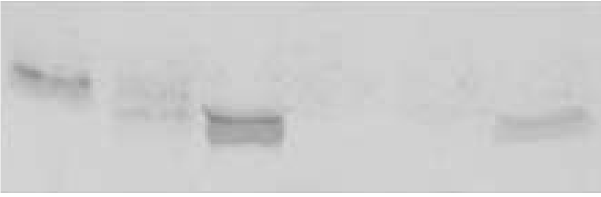

4. From 20 to 31

Medium

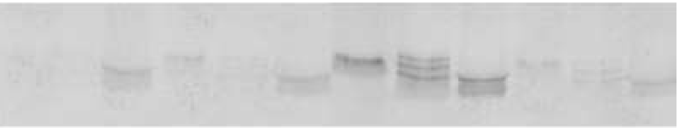

Figure 6 mab9c5

1. Left side blot represents lanes 2 to 11 of figure 6
2. Right side blot represents lanes 12 to 19 of figure 6

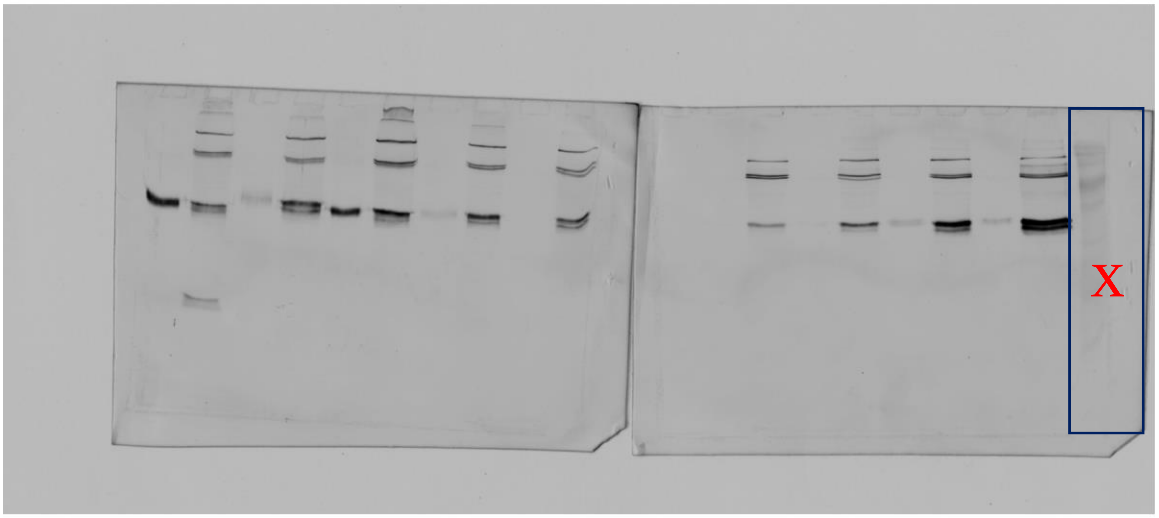

Figure 6 mab220

1. Left side blot represents lanes 2 to 12 of figure 6
2. Right side blot represents lanes 13 to 19 of figure 6

'X' represents not used lanes

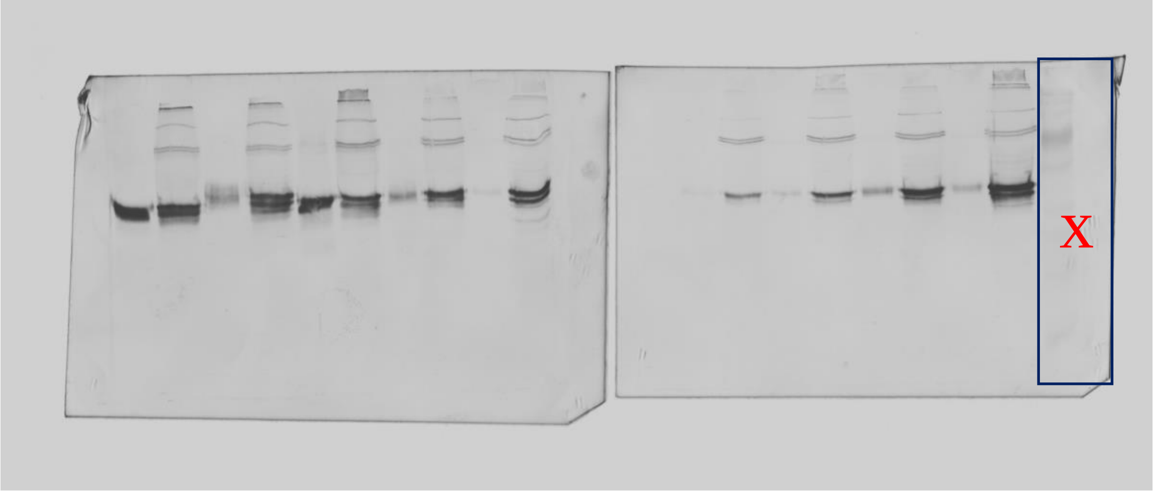

Figure 7 Cells 5x

1. Left side blot Boxed red – Lanes 5 to 10 of figure7 (cells)
2. Right side upper blot - Lanes 11 to 16 of figure7 (cells)
3. Below left side blot represents lanes 17 to 22 of figure7 (cells)
4. Below right-side blot represents lanes 23 to 31 of figure7 (cells)

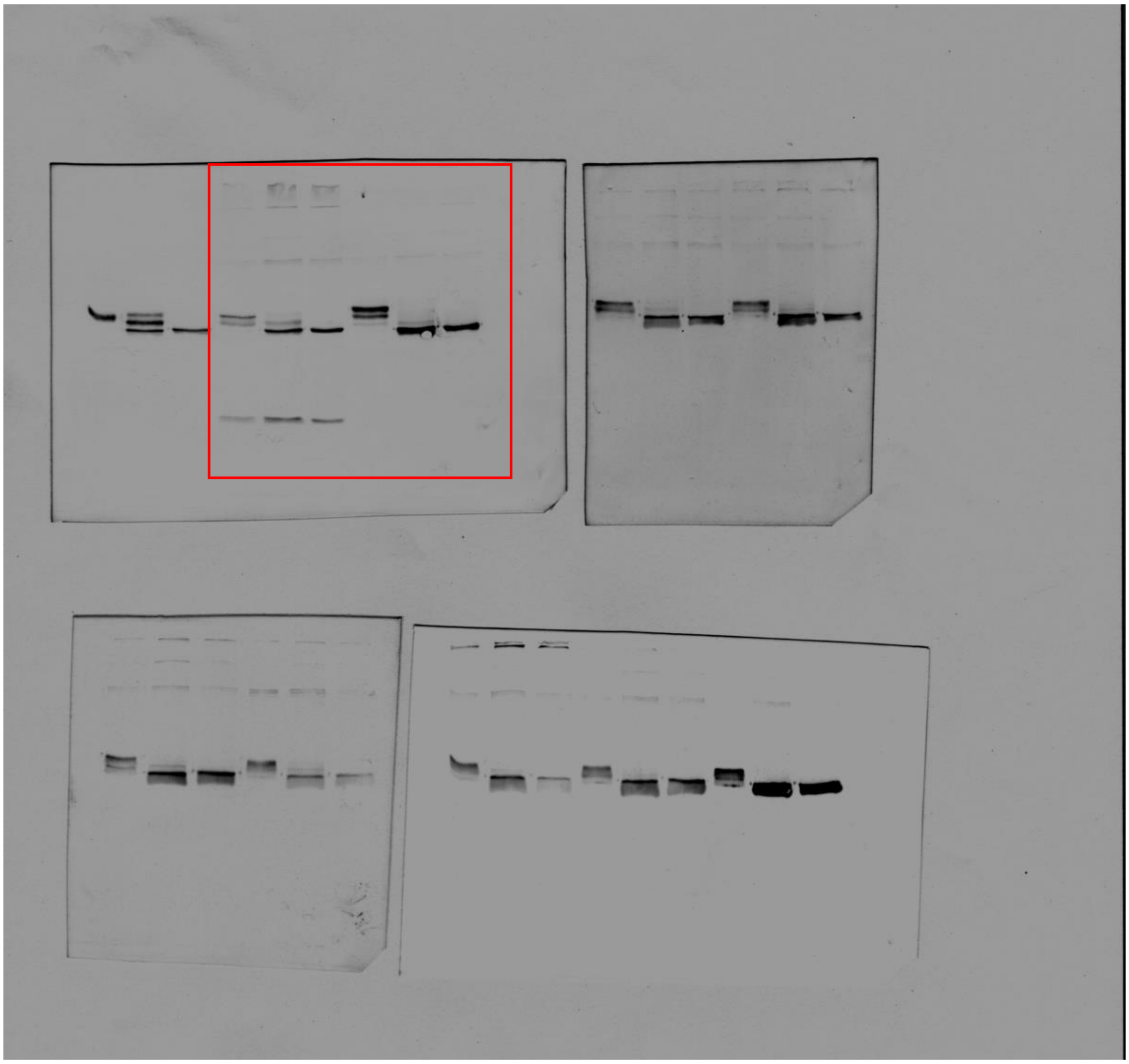

Figure 8 4x

Left side blot (2 to 11 lanes of figure 8) right side blot (12 to 21 lanes of figure 8)

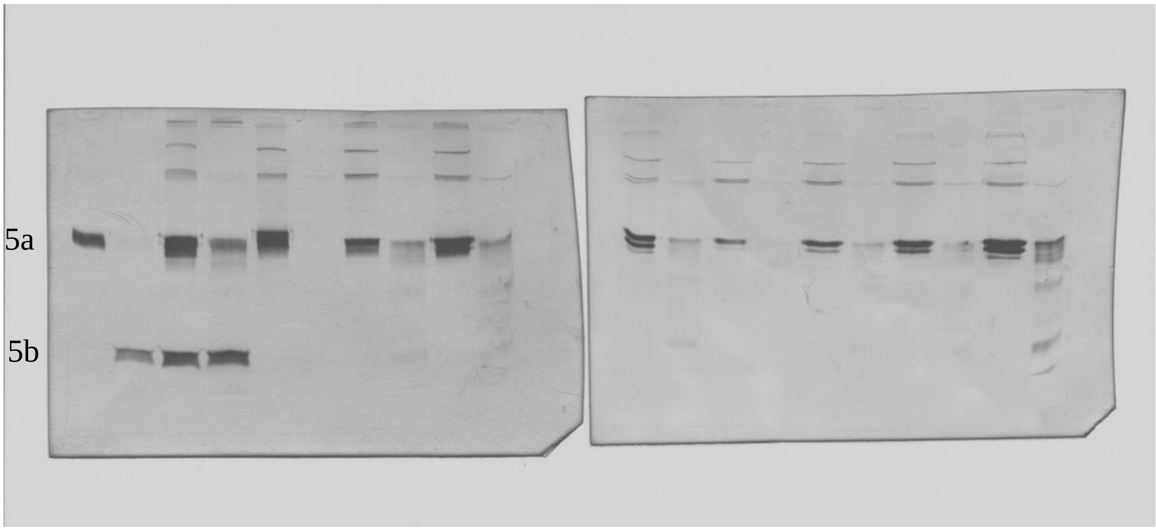

Supplement: S1 Fig — (PDF) [file pone.0230052.s001.pdf]
