# Supplementary material for: Characterisation of ACP5 missense mutations encoding tartrate-resistant acid phosphatase associated with spondyloenchondrodysplasia
Source: PLoS One. 2020 Mar 26;15(3):e0230052. doi: 10.1371/journal.pone.0230052 (PMC7098635; doi:10.1371/journal.pone.0230052)
Supplement: Attachment — Submitted filename: Response to Reviewers.docx [file pone.0230052.s002.docx]

**Response to reviewers**

The authors profusely thank the reviewers’ for their constructive and valuable criticism which we strongly believe making our draft paper more refined and may be acceptable at this time. We earnestly apologize for typos, errors and delay in responding to review as our labs are being shifted from one campus to the other which made “our hands tied” to respond to all the recommendations. As the authors emanate from a non-English speaking country, we still learning and refining our way of writing. Hope we will get better in the future and certainly referees’ recommendations are tremendous helpful to refine our paper. This time we have given our manuscript to an English professor who was kind enough to correct the grammatical errors and typos. Figures which are ambiguous and “not scientifically talking back” are either removed or renumbered.

Again, the authors thank the referees and we feel at this time, this revised manuscript should be acceptable in Plos.One, which we consider as highest honor to publish from the east end. We will be happy to modify further if needed. Details on SPENCD disorder related abnormalities are coming up sharply in the last decade.

**Referee 1:**

Authors profusely thank the Referee 1 for the constructive and immensely helpful criticisms.

1. We surely apologize for the conspicuous errors in the Results section for placing the figure numbers upside down. As recommended we now either correctly and sequentially place or change the figure numbers. Entire Result section was rewritten eliminating unwanted details so that both figures and results will coincide with each other.

2. As rightly pointed out by the Referee, figure legends are rewritten so that they are devoid of experimental details. Experimental repeats were also mentioned.,

3. As recommended the figures 6a and 6b were renumbered as 6 and 7.

4. With regard to the observation of similar levels of intracellular TRACP mutant proteins.

We have discussed in appropriate section with regard to the similar levels of TRACP mutant proteins (Results and Discussion as well). First, we did all the experiments with stably transfected cell lines which took a large amount of time to perfect in between experiments. Realizing the cell number variations in different experiments may generate spurious values, we culture exact same number of cells per flask by counting both before and after culturing. This technique largely eliminated the quantitative variations between experiments, and we were able to do this as our personnel are fully trained and routinely do these techniques for hematology labs.

5. As recommended the discussion was sharply shortened and now it should be sufficing.

6. Most of our typos and grammatical errors were corrected with the help of an English professor. As the authors are from a non-English speaking country we still evolving and hope umbrage will not be taken for this inability and deficiency.

**Referee 2:**

1. Overall aim of the paper is now mentioned in the introduction.

2. As recommended excessive explanation in the Results section was eliminated in toto and discussion section was also modified and shortened.

3. Relationship between SPENCD and TRACP was also elaborated in the Introduction.

4. Molecular weight of TRACP isoforms 5a and 5b in the Western blots were indicated now. Putting together to make a meaningful picture was a herculean task for us and therefore we have used professional help to put together our multiple pictures (using Adobe Photoshop by Ms. Barbara J, please *vide* acknowledgement). We normally run standard proteins markers and to put together multiple runs at different points with standard marker proteins (which have small difference in migration and therefore they were removed). Now we marked the molecular weights of 5a and 5b proteins as they are the only primary bands of interest.

Thanking you.
