# Supplementary material for: Characterisation of ACP5 missense mutations encoding tartrate-resistant acid phosphatase associated with spondyloenchondrodysplasia
Source: PLoS One. 2020 Mar 26;15(3):e0230052. doi: 10.1371/journal.pone.0230052 (PMC7098635; doi:10.1371/journal.pone.0230052)
Supplement: Attachment — Submitted filename: Response to Reviewers.docx [file pone.0230052.s003.docx]

To	Date: 2/11/2020

Dr. Sakamuri V. Reddy,

Academic Editor,

PLOSOne

Ms. PONE-D-19-20746R1 - Revised manuscript

Dear Dr. Reddy,

Enclosed, please find our revised manuscript entitled “Characterisation of *ACP5* Missense Mutations Encoding Tartrate-Resistant Acid Phosphatase associated with Spondyloenchondro- dysplasia by Janani Ramesh *et al* for publication in the PLOSOne.

I apologize for the delay in sending this revised manuscript. As mentioned in my earlier correspondence, our lab shifting issues to move into a different floor with equipments and lab materials made me almost immovable. Nevertheless, with the help of my co-authors, we have sincerely attempted to address all of the reviewers' concerns and incorporate their suggestions especially on English. We are extremely thankful to those two reviewers for their objective critique of the manuscript and pointing out the salient deficiencies. We are also extremely gratified to note that addressing these deficiencies *significantly increased the quality of our paper.*

As pointed out by the reviewers, a major weakness of the paper is the English and we believe that we have sufficiently addressed this issue in this revised version. One of the senior professors of our University of Louisville (Prof. Rif S. El-Mallakh) has immensely helped with the English of this paper and for that gratitude we have included him as an author. Figures which are ambiguous are either removed or modified. Again the authors thank the referees profusely and we hope that this revised manuscript may be acceptable at this time.

We also hope that the changes we made with the approval of the reviewers and yours should be acceptable at this time. We eagerly look forward to a favorable decision. Our specific responses to the reviewers’ comments are attached separately (point-by-point).

Thank You.

Sincerely,

Dr. Bhuvarahamurthy Venugopal.

**PONE-D-19-20746R1**

The authors profusely thank the reviewers for their constructive and valuable criticism which we strongly believe has enhanced the quality of the revised paper. We apologize for all errors in the earlier version. As recommended, we have now revised our manuscript according to the reviewers’ suggestions and we have tried our best by giving detailed responses to each of the reviewers’ questions point-by-point.

**Reviewer #1: There is only slight improvement in the revised manuscript.**

**Major issues:**

**1) Authors have not provided point-by-point response in detail. Very casual approach is taken by authors in writing of the** **response to reviewers’ comments.**

**Reply to the Reviewer 1:** We apologize for the conspicuous errors in the Results section. We have added more information and rewritten the results section in better English.

**2) There is only slight improvement in English writing in the manuscript.**

**Reply to the Reviewer 1:** As recommended, we have now corrected the entire manuscript especially with regard to English and grammar.

**3) However, the scientific language is still very poor throughout the manuscript, particularly the authors have not used proper** **scientific terms and framing of the sentences and grammar. It appears that manuscript is not properly edited by senior** **authors.**

**Reply to the Reviewer 1:** As recommended several changes have been made throughout the manuscript with proper scientific terms and framing the sentences in better English.

**3) Result section is still very poor. Results of all the experiments are not written in details. The sub-figures are not cited in the** **text. Although, there are several sub-figures in many figures such as Figures 3, 4, 5 and 9, authors have just written there** **numbers.**

**Reply to the Reviewer 1:** Results section has been drastically modified and details added wherever necessary. As recommended, Discussion was also drastically shortened. The sub-figures have been cited in the text. We have revised the figure numbers and attached as individual files as recommended.

**4) Many Figures Legends are incomplete. e.g. In Figure 1 Legend, it is mentioned that missense mutations are shown below** **the WT sequences. This is not reflected in actual figure. Similarly, there are many mistakes in other Figures legends.**

**Reply to the Reviewer 1:** We have revised and rewritten the figure legends as recommended.

Figure 1 was carefully revised to reflect the details of TRACP.

**Reviewer #2:**

**Characterization of Acp5 Missense Mutations Encoding Tartrate-Resistant Acid**

**Phosphatase Associated with Spondyloenchondrodysplasia** **The MS has been revised as suggested, and the authors corrected the crucial issues.** **It can be accepted for publication**

**Reply to the Reviewer 2:** We thank you for your suggestions.

**Reviewer #3:**

This article attempts to characterize the eight already reported missense mutations in Acp5 which is associated with spondyloenchondrodysplasia (SPENCD). The authors identified that the presence of mutants TRACP proteins are unable to induce enzymatic activity. Further, except T89I and M264K mutant proteins, others are in denatured precursor forms. The authors concluded that understanding the structural and functional aspects of mutant TRACP could lead to better understating of immune response and bone metabolism as well as targeting drug development. The concept of the paper is relevant however there are many concerns that should be addressed before publication. The authors should follow the scientific writing format rather than common words and sentences. The whole manuscript should be corrected for details, clarity, and grammar.

**Major issues:**

**1) In Abstract: RT-qPCR analysis is mentioned but there is no such figure and**

**result in appropriate sections.**

**Reply to the Reviewer 3:** Since the contribution by RT-qPCR study is minimal we have removed this section from our paper and changed all sections accordingly.

**2) Methods section written poorly, refer the articles and follow the formats. For**

**instance, Western blotting is written without clarity and details. Some of the Methods**

**written like results that should be avoided.**

**Reply to the Reviewer 3:** As recommended we have revised our Methods section and added more details to Western blot technique. Results reported in Methods section have been removed as per suggestion.

**3) It is not appropriate to write a sentence like this in the result section “Other salient features of TRACP are described in** **Figure 1 legend”. Provide more details about TRACP enzyme in this section.**

**Reply to the Reviewer 3:** As suggested, we have provided details about TRACP enzyme in this Results section with Figure 1.

**4) “The steady state transcription…..10 fold lower compared to WT”. Thereis no corresponding figures/results in this manuscript.**

**Reply to the Reviewer 3:** As RT-qPCR has been removed in this revised version, sentences related to this work have also been removed from other sections of this resubmission.

**Minor issues:**

**1) In Abstract:** **At the end, summarize or conclude the work and it should exactly reflect the results. Then write what it could** **be useful to, such as drug development and underlying mechanisms, etc.**

**Reply to the Reviewer 3:** As recommended, we have completely rewritten the Abstract..

**2) In Abstract: “Certainly, determining the structure functional relationships in TRACP will expand….” Should modify to** **“****Determining the structural and functional relationships of TRACP may expand… “**

**Reply to the Reviewer 3:** As recommended, we have modified these sentences as “Determining the structural and functional relationships of TRACP may expand…”.

**3) An unscientific sentence like this and many others throughout the manuscript should be modified “TRACP has many** **functions in vitro and its biological role in bone resorption and immune responses is becoming clearer”. What exactly authors**

**want to convey with this sentence “TRACP has many functions in vitro….”**

**Reply to the Reviewer 3:** As recommended, we have modified this sentence. Please see our revised manuscript.

**4) “Therefore, serum TRACP5 may serve as a marker for systemic macrophage number….” Is it possible to count the macrophage number using serum TRACP5a level? Similarly, isoform 5b release and osteoclast number?? Clarify both statements.**

**Reply o the Reviewer 3:** We have modified the sentence with clarity in this revised version.

**5) Mentioned in this sentence that “We and others have shown earlier [9, 10] that mutations…” but there is one other’s paper** **cited, add more reference or modify.**

**Reply to the Reviewer 3:** As recommended, we have now added more references here.

**6) Follow the same format for gene name, ACP5 or Acp5.**

**Reply to the Reviewer 3:** We apologize for this inconsistency. We have consistently followed the same pattern of human gene nomenclature. Human gene should be in caps and we have changed accordingly throughout the manuscript.

**7) Add references to these sentences and many others "More specifically, ….TRACP enzyme”, “Other salient** **features…. calcification”**

**Reply to the Reviewer 3:** As suggested, we have now added references to these sentences.

**8) “Transfection of synthetic genes” This subtitle should be written like this “Transformation of mutant genes and subcloning”** **and describe the methods in the text.**

**Reply to the Reviewer 3:** As directed, the subtitle has been changed accordingly.

**9) “Transient transfection and generation of stable cell line” This should be a subtitle for the next paragraph (from this** **sentence onwards “HEK-293 cells were transfected with……recommended procedures.”**

**Reply to the Reviewer 3:** As suggested, the subtitle has been inserted in its place.

**10) What is the loading control used for Western blot analysis? Explain**

**Reply to the Reviewer 3: Since there are no other proteins present in the blot other than our electrophoretically homogeneous TRACP proteins, we treat this protein as loading control. Also the electrophoresis combs are not sufficient enough to increase the number of samples.**

**11) Figures should be labeled appropriately. For instance, Fig 2, TRAP staining and Mab220.**

**Reply to the Reviewer 3:** We thank the reviewer for the comments. As rightly pointed out by the Referee, figures have been rewritten appropriately, and the figure legends have also been rewritten so that they are devoid of experimental details.

**12) Y-axis scale difference is high between min and max in many bar graphs, therefore if it appropriate to represent the graph with the broken y-axis to show the lower values.**

**Reply to the Reviewer 3:** We have revised the graphs as much as the Software package allows.

**13) The results section should be written clearly with details.**

**Reply to the Reviewer 3:** We have corrected the Results section with clarity now.
